# Medium Chain Fatty Acids Are Selective Peroxisome Proliferator Activated Receptor (PPAR) γ Activators and Pan-PPAR Partial Agonists

**DOI:** 10.1371/journal.pone.0036297

**Published:** 2012-05-23

**Authors:** Marcelo Vizoná Liberato, Alessandro S. Nascimento, Steven D. Ayers, Jean Z. Lin, Aleksandra Cvoro, Rodrigo L. Silveira, Leandro Martínez, Paulo C. T. Souza, Daniel Saidemberg, Tuo Deng, Angela Angelica Amato, Marie Togashi, Willa A. Hsueh, Kevin Phillips, Mário Sérgio Palma, Francisco A. R. Neves, Munir S. Skaf, Paul Webb, Igor Polikarpov

**Affiliations:** 1 São Carlos Physics Institute, University of São Paulo, São Carlos, Sao Paulo, Brazil; 2 Diabetes Research, Methodist Hospital, Houston, Texas, United States of America; 3 Institute of Biosciences, São Paulo State University, Rio Claro, Sao Paulo, Brazil; 4 Department of Pharmaceutical Sciences, University of Brasilia, Brasilia, Brazil; 5 Chemistry Institute, State University of Campinas, Campinas, Sao Paulo, Brazil; University of Bari & Consorzio Mario Negri Sud, Italy

## Abstract

Thiazolidinediones (TZDs) act through peroxisome proliferator activated receptor (PPAR) γ to increase insulin sensitivity in type 2 diabetes (T2DM), but deleterious effects of these ligands mean that selective modulators with improved clinical profiles are needed. We obtained a crystal structure of PPARγ ligand binding domain (LBD) and found that the ligand binding pocket (LBP) is occupied by bacterial medium chain fatty acids (MCFAs). We verified that MCFAs (C8–C10) bind the PPARγ LBD *in vitro* and showed that they are low-potency partial agonists that display assay-specific actions relative to TZDs; they act as very weak partial agonists in transfections with PPARγ LBD, stronger partial agonists with full length PPARγ and exhibit full blockade of PPARγ phosphorylation by cyclin-dependent kinase 5 (cdk5), linked to reversal of adipose tissue insulin resistance. MCFAs that bind PPARγ also antagonize TZD-dependent adipogenesis *in vitro*. X-ray structure B-factor analysis and molecular dynamics (MD) simulations suggest that MCFAs weakly stabilize C-terminal activation helix (H) 12 relative to TZDs and this effect is highly dependent on chain length. By contrast, MCFAs preferentially stabilize the H2-H3/β-sheet region and the helix (H) 11-H12 loop relative to TZDs and we propose that MCFA assay-specific actions are linked to their unique binding mode and suggest that it may be possible to identify selective PPARγ modulators with useful clinical profiles among natural products.

## Introduction

Peroxisome proliferator activated receptors (PPARs α, β/δ and γ) are ligand-dependent transcription factors that are prominent targets for pharmaceutical development. Thiazolidinediones (TZDs) act through PPARγ to elicit increased sensitivity to insulin in type 2 diabetes mellitus (T2DM) and reduce inflammation in arteries [1]. Unfortunately, TZDs also exhibit deleterious effects on fat accumulation, fluid retention and bone density and increase risk of heart failure, indicating a need for new selective PPARγ ligands with improved clinical profiles [1]–[3].

In addition to TZDs, PPARγ binds natural lipophilic molecules, including long chain fatty acids (FAs), oxidized or nitrated FAs, prostaglandins and arachidonic acid derivatives [4], [5] but possible selective activities of these compounds have not been assessed. Some reports suggest that PPARγ ligands with weak partial agonist activity relative to TZDs exhibit beneficial effects equivalent to strong agonists, with fewer harmful side effects [6]–[9]. At least some insulin sensitizing effects of TZDs mediated by PPARγ do not require full agonist actions; TZDs block cyclin-dependent kinase 5 (Cdk 5) mediated phosphorylation of PPARγ ser273, which reduces expression of key adipokines in fat cells [10]. Improved knowledge of relationships of PPARγ ligand binding modes and relationships to partial agonism and secondary modifications could help us develop selective ligands that act as safer PPARγ modulators.

PPARs are nuclear hormone receptors (NRs) [11]. Like other NRs, PPARs bind specific DNA response elements (PPREs), usually as a heterodimer with retinoid X receptor, and modulate transcription of nearby genes by recruiting coregulator complexes [3], [12]. Agonists alter target gene expression by binding the ligand binding pocket (LBP) in the core of the ligand binding domain (LBD). This, in turn, induces conformational changes which result in increased stability of the entire LBD and altered position and dynamics of LBD C-terminal helix (H) 12, with the latter effect remodeling of a cofactor binding site on the LBD surface to favor binding of coactivators over corepressors [12].

Despite similarities between actions of PPARs and other NRs, PPAR LBPs exhibit distinctive characteristics [13], [14]. PPAR LBPs are large (≈1300 Å^3^) Y- or T-shaped cavities which are partly open to the LBD surface and only partially filled by TZDs or other known ligands, different from LBPs of thyroid hormone receptors (TRs), steroid receptors and other NRs which tend to be small (≈500–600 Å^3^) with ligand tightly enclosed [11]. Further, PPARs exhibit multiple ligand binding modes; different PPARγ ligands bind at different locations in the LBP and the PPARγ LBP can accommodate two ligands at the same time [4]. Strong PPARγ agonists such as the TZDs rosiglitazone (rosi) and pioglitazone (pio) directly contact H12 whereas partial agonists bind towards the base of the Y-shaped LBP, do not contact H12 and stabilize the β-sheet/H2-H3 region thereby inhibiting cdk5 phosphorylation [10], [15]–[17].

Here, we crystallized PPARγ LBD in a form that diffracts to relatively high resolution in the absence of exogenous ligands. The structure resembles previous liganded and unliganded PPARs [4], [18] but close investigation reveals three saturated medium chain fatty acids (MCFAs) occupy the LBP at the same time and mass spectroscopic analysis suggests that these are predominantly nonanoic acid (NA, C9) with a smaller amount of octanoic acid (OA, C8). C8–C10 MCFAs are PPARγ essentially partial agonists, but exhibit assay-specific variations in activity relative to TZDs and MCFAs that bind PPARγ block TZD-dependent adipogenesis. A recent paper also revealed that a C10 MCFA acts as a modulating ligand of PPARγ, but this group found a single molecule of C10 binds the pocket and rationalized partial agonist activity in terms of weak H12 stabilization [19]. Our X-ray crystal structure B-factor analysis coupled to molecular dynamics (MD) simulations [20] suggests that diverse agonist/partial agonist behaviors may be linked to the tripartite MCFA binding mode and raise the intriguing possibility that selective PPAR modulators with useful context-selective properties may be identified among natural products. We discuss the possibility that MCFAs are natural PPAR ligands.

## Results

### Three Ligands in the PPARγ LBP

**Figure 1:**
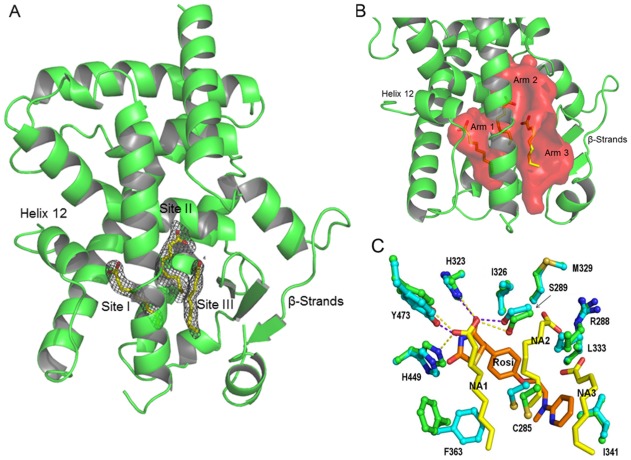
PPARγ binds three MCFAs. (**A**) Structure of the PPARγ LBD chain A subunit showing positions of MCFA electron densities in the LBP; NA1 (site I), NA2 (site II) and NA3 (site III). (**B**) LBP (in red) with its three arms filled with MCFA (**C**) Overlaid views of the PPARγ LBP in the presence of NA (PPARγ green, ligand yellow) and Rosiglitazone (PPARγ blue, ligand transparent pink) revealing ligand binding modes. **C**) Radiolabeled ligand displacement assay. Bacterially expressed PPARγ LBD was incubated with radiolabeled rosiglitazone +/_ cold competitors as indicated.

We obtained PPARγ LBD crystals without exogenous ligand and subsequent X-ray structural analysis revealed that they diffracted to relatively high resolution (2.1 Å, Table S1). The new PPARγ structure closely resembles previous PPARγ LBD structures (Fig. 1A) [4], [18]. The LBD crystallized as a homodimer (A and B-chains) with the A-chain exactly corresponding to the canonical active NR LBD fold with H12 in an active position (Fig. 1A) and the B-chain in an inactive conformation with H12 protruding away from the molecule (Fig. S1). More surprisingly, close investigation of the PPARγ A-chain LBD revealed three elongated and well-defined ligands in the LBP (Figs. 1A, B). Electron density is strong, consistent with high occupancy. Two similar ligands were present in the B-chain LBD but these are poorly resolved, similar to previous descriptions of ligand binding to PPARγ B-chains [4]. To our knowledge, this is the first time that exogenous ligands have been shown to occupy the LBP of a putative apo-PPARγ structure.

### MCFAs associate with PPARγ LBP

**Figure 2:**
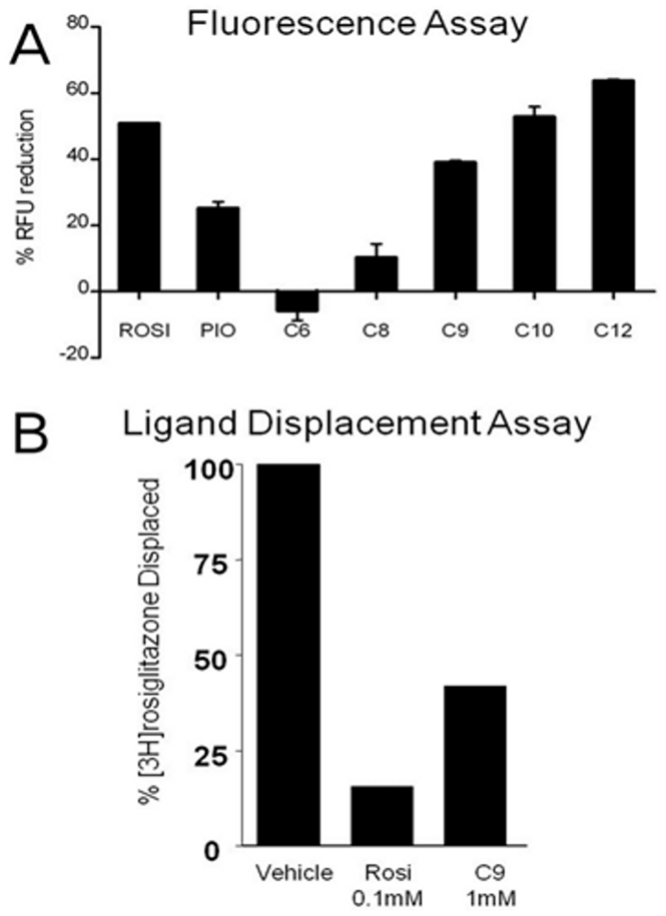
C8–C10 MCFAs bind and Stabilize PPARγ LBD *in vitro*. Analysis of MCFA binding to PPARs by dye binding assays, performed with purified PPARγ LBD and 0.1 mM TZDs or 1 mM FAs at room temperature. The figure shows the percentage reduction in fluorescence versus unliganded PPARγ treated with vehicle for different MCFAs. Briefly, this technique measures interactions of fluorescent dye (ANS) with exposed hydrophobic protein surface and, since hydrophobic amino acids are buried in the core of folded domains by hydrophobic effect, provides an index of structural stability. Apo-PPARγ LBD exhibits high fluorescence, suggestive of partial unfolding. TZDs (rosiglitazone, rosi, pioglitazone, pio, 0.1 mM) reduce fluorescence index, indicative of ligand-induced folding. In parallel, decreases in fluorescence index were obtained with OA (C8), NA (C9), DA (C10) and LA (C12) but not HA (C6) (all at 1 mM final concentration). Fluorescence was monitored in a standard Q-PCR machine (Phillips Lightcycler).

The ligands in the PPARγ LBP are bacterial MCFAs. Mass spectroscopic analysis revealed that MCFAs were associated with our purified PPARγ LBD preparations and that these are predominantly nonanoic acid (C9:0, NA, 80%), with smaller amounts of octanoic acid (C8:0, OA, 20%) (Fig. S2). There is no obvious source of these ligands in purification reagents or buffers and it is therefore likely that they are bacterial in origin and persist throughout purification. Accordingly, we used the major ligand associated with the PPARγ preparations, NA (C9), for X-ray structure model building and found that it fits well with observed electron densities in the LBP (Fig. 1B). Added MCFAs (C8–C10, but not C6) bind and stabilize purified PPARγ LBD in a modified differential fluorescence scanning (DSF) assay [21], which detects ligand-dependent reductions in solvent-exposed protein hydrophobic surface and is indicative of protein folding (Fig. 2A). Moreover, NA (C9) displaced radiolabeled Rosi from bacterially expressed PPARγ LBD, albeit with much lower potency than unlabeled Rosi (Fig. 2B). Thus, MCFAs are *bona fide* PPARγ interacting compounds, albeit weak binders. Longer chain saturated fatty acids (C14–C18) are known to bind PPARγ [22], but this report, coupled to a recently published report [19] establishes MCFAs as PPARγ interacting ligands.

### MCFA Binding Modes

The trimeric MCFA ligand binding mode is unprecedented (Fig. 1B). Each MCFA binds one arm of the PPARγ A-chain LBP and, together, the three molecules occupy about 52% (≈630 Å^3^) of LBP total volume (Fig. 1B, C). While it was previously shown that PPARγ LBP can accommodate two copies of the same ligand [4] it has never been shown that three copies of the same ligand can simultaneously occupy the PPARγ LBP.

Each NA occupies one arm of the Y-shaped pocket (Fig. 1B). NA1 is within the polar arm, close to H12, and makes extensive contacts with LBP amino acids. The carboxylate interacts with Y473 on the inner H12 surface (2.90 Å), H323 (2.92 Å), H449 (2.75 Å) and S289 (3.05 Å) and the hydrophobic tail interacts with a surface formed by I281, F282, L353, F363, M364 and L453. NA2 and NA3 make few direct contacts with protein. This position is similar to that occupied by the single decanoic acid molecule (C10) located in the recently published PPARγ:C10 MCFA structure [19]. NA2 occupies a site between H1, H3 and H4/5 with the carboxylate group in contact with R288 (3.2 Å) on H3 and the tail stabilized by hydrophobic interactions with A292, I296, M329 and L330. NA3 is close to the base of the Y, between H3 and the β-strands. Like NA2, the NA3 carboxylate also interacts with the R288 side chain (3.87 Å, Nε) and also binds the main chain at L340 (3.2 Å) and the NA hydrophobic tail interacts with I341 and C285.

LBP amino acids that contact each NA ligand have all previously been shown to contact other PPARγ interacting compounds [4], [5], [18]. NA1 binds Y473 on the inner face of H12, also important in TZD binding, whereas NA2 and NA3 carboxylates interact with R288, which does not bind TZDs but does bind oxidized FAs 13-HODE and 9-HODE, nitrated FAs and synthetic partial agonists. Comparisons of the PPARγ+MCFA structure with PPARγ-TZD structures reveal differences between TZD and MCFA contact modes (Fig. 1C). Most obviously, the Phe363 (H7) side chain binds the NA1 aliphatic chain but adopts an opposite orientation in PPARγ+rosi structures and is not involved in ligand contact. There are also shifts in positions of Ser289 (H3), His449 (H11), Tyr473 (H12) and other residues. However, the main difference the PPARγ+MCFA structure and PPARγ+TZD structures is that all arms of the pocket are occupied by MCFAs, whereas TZDs only contact residues in two arms of the Y.

MCFA interactions with the PPARγ chain B LBP partly resemble those of chain A (Fig. S1). The two MCFAs occupy positions that approximately correspond to NA2 and NA3 in Chain A. However, the NA2 aliphatic chain adopts a slightly different position in Chain B, and the NA3 aliphatic chain appears highly disordered. More importantly, no ligand occupies the NA1 position at the inner surface of H12. This implies that MCFA binding at the NA1 position is coupled to H12 packing (Discussion).

### MCFAs are pan-PPAR Partial Agonists and Display Assay-Specific Partial PPARγ Agonist Effects

**Figure 3:**
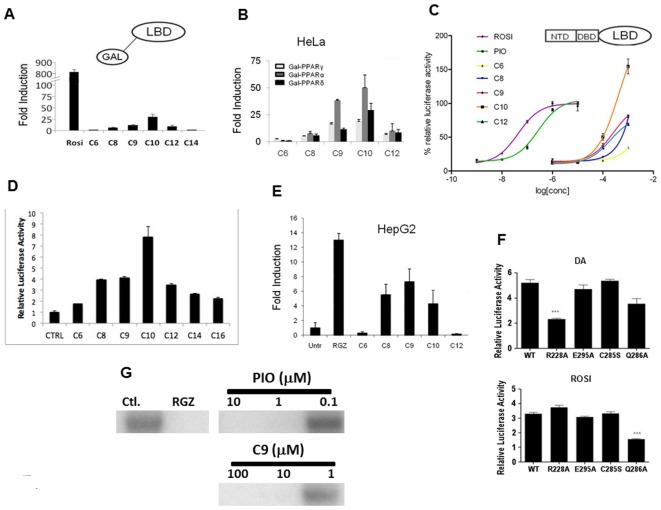
MCFAs exhibit assay-specific differences in agonist efficacy. (**A**) Transfections with GAL-RE luciferase reporter + Gal-PPARγ LBD expression vector and treated with 10 µM Rosi or 1 mM MCFAs), standard errors derived from quadruplet points and experiments repeated >3 times. (**B**) MCFAs are pan-PPAR activators. Comparison of abilities of MCFAs (1 mM) to activate Gal-PPAR LBDs in HeLa cell transfection assays. (**C**) Results of transfections (HeLa cells; DR1 luciferase) +/− PPARγ expression vector showing dose responses, standard errors derived from quadruplet points and experiments repeated >3 times. Data expressed as % induction relative to saturating TZDs. (D) Transfections to compare MCFA and LCFA activities at a DR-1 element, data expressed as fold FA induction relative to control. **E**) MCFAs are PPAR partial agonists in HepG2 cells. Results of transfection analysis with full length PPARγ and a PPRE driven luciferase reporter gene +/− 1 mM MCFA, as in Fig. 4D. (**F**) Transfections +/− PPARγ or PPARγ mutant expression vector and treated with Rosi or DA. Data expressed as fold induction by MCFAs relative to untreated. (**G**) An MCFA blocks PPARγ LBD phosphorylation by cdk5 *in vitro*; SDS-PAGE gel to reveal ^32^P-labeled PPARγ LBD, samples include LBD with no ligand or with increasing Rosi or NA (C9) (0.01, 0.1, 1 mM).

MCFAs (1 mM) behave as partial pan-PPAR agonists in transfections. MCFAs were very weak partial agonists at a GAL-PPARγ LBD fusion, which is highly AF-2 dependent (Fig. 3A). Here, C6 (which does not bind PPARγ) failed to activate transcription but longer MCFAs that do bind PPARγ (C8, C9, Decanoic acid, DA C10 and Lauric acid, LA C12) elicited low partial agonist activity, with C10 most effective (about 3–5% of rosi in this assay). Similar activation patterns were also seen with GAL-PPARα and –PPARδ fusions (Fig. 3B). Interestingly, MCFAs were more efficient partial agonists with full length PPARs (Fig. 3C, D). Here, OA (C8), NA (C9) exhibited up to 70% of TZD activity at a PPRE-regulated reporter in HeLa cells and DA (C10) slightly stronger than TZDs (Fig. 3C) and LCFAs (C14, C16) in this cell type (Fig. 3D). In other cell types, including HepG2, effects were somewhat weaker and C8–C10 MCFAs activated transcription with about 50% of the activity of rosi (Fig. 3E). MCFAs are not potent agonists; whereas 1–10 µM TZDs were sufficient for maximal PPARγ activation, C8–C10 FAs only exhibited activity in the 100 µM-1 mM range (Fig. 3C). Effects of PPARγ LBP mutations are consistent with predictions about binding mode derived from X-ray structures (Fig. 3F). Mutation of R288, which interacts with MCFAs at sites II and III but not with rosi or other TZDs (PPARγR288A) or with MCFAs at site I, compromised PPARγ response to DA, but not rosi. Conversely, an amino acid implicated in TZD interaction but not MCFA interaction (PPARγQ286A) was needed for rosi response but was dispensable for DA response. Mutation of nearby residues that do not interact with MCFAs or rosi (PPARγE295A and C285S) did not affect responses to either ligand. Finally, MCFAs strongly inhibited cdk5-dependent phosphorylation of PPARγ LBD preparations *in vitro*. NA (C9) inhibited cdk5 dependent phosphorylation of bacterially expressed PPARγ LBD preparations as efficiently and potently as rosi (Fig. 3G).

### MCFAs that Bind PPAR Antagonize Adipogenesis

**Figure 4:**
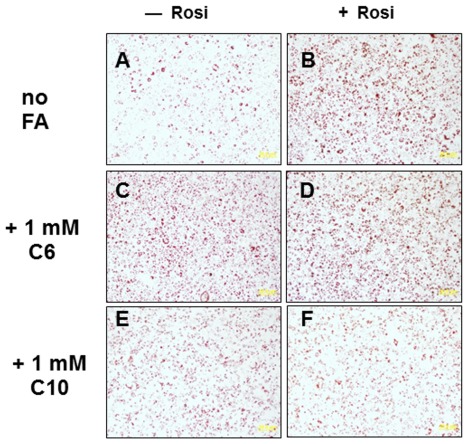
MCFAs that bind PPARγ block TZD-dependent 3T3-L1 cell differentiation. 3T3 fibroblasts were induced to differentiate with Insulin, Dexamethasone and Isobutylmethylxanthine −/+ 100 nM rosi (**A, C, E** versus **B, D, F**), 1 mM HA C6 (**C, D**) or 1 mM DA C10 (**E, F**). Adipogenesis was tested by Red Oil O staining and results showed that DA C10, but not HA C6, is weakly adipogenic and blocks rosi effects.

As previously documented, we found that MCFAs were influenced adipoegenesis [19], [23] but also showed that MCFAs that bind PPARγ can antagonize rosi effects. HA C6 (1 mM), which does not bind PPARγ, triggered similar levels of fat droplet accumulation to rosi (compare Fig. 4A, 4B and 4C) and failed to antagonize rosi response (Fig. 4D). By contrast, DA (C10, 1 mM) was weakly adipogenic (compare Fig. 4E to Fig. 4A) but strongly antagonized rosi response. Similar results were also obtained with OA (C8, not shown). Thus, an MCFA that does not bind PPARγ cannot block rosi-dependent adipogenesis, whereas MCFAs that do bind PPARγ are anti-adipogenic, raising the possibility that some anti-adipogenic actions of MCFAs may be PPARγ-dependent (see Discussion).

### MCFAs and Rosi Induce Differences in PPARγ External Stability

Given assay-specific variations in efficacy of MCFAs (weak AF-2 partial agonist, stronger partial agonist with full length PPARγ and full agonist in blockade of ser273 phsophorylation), we set out to compare MCFA effects on PPARγ conformation with TZDs. This required us to obtain a PPARγ+rosi structure in the same space group as our PPARγ+NA structure (2.5 Å resolution, Table S1) to compare NA and rosi influences on PPARγ organization without confounding effects of differences in crystal packing [18].

**Figure 5:**
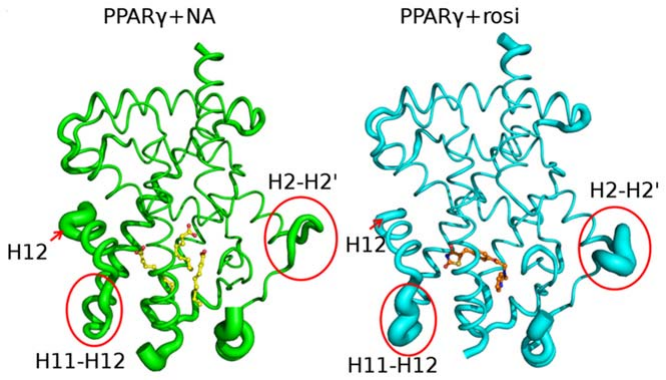
Rosiglitazone and MCFAs stabilize PPARγ LBD in distinct ways. Variations in crystallographic B-factors represented as width of the backbone trace in PPARγ-NA (left) and PPARγ+rosi (right).

PPARγ+NA and PPARγ+rosi structures exhibit identical overall fold and dimer organization. However, there are differences in crystal structure B-factors in the presence of rosi and MCFAs; these provide an index of relative mobility of different parts of the protein in the crystal lattice (Fig. 5). H12 appears better packed against the LBD surface with rosi (arrow) than NA. By contrast, the loop between H11 and H12 and the H2-H3/β-strand regions appear more ordered with NA than rosi (circles). Both regions are important in PPARγ function, changes in H11-H12 loop structure have been implicated in H12 dynamics [24] and, as mentioned above, partial PPARγ agonists preferentially stabilize the β-strand region [15]. Further, the H2-H3 loop region overlaps ser273, the target of cdk5 phosphorylation, which is efficiently blocked by MCFAs. Thus, the two ligand types exhibit differential effects on PPARγ LBD external stability.

### MCFA Chain Length Influences H12 Dynamics

To better understand between MCFA binding mode and activity we performed MD simulations based on the PPARγ+NA X-ray structure in a shell of water and ions to simulate aqueous conditions [20]. The technique allows us to predict and observe ligand and protein dynamics over short times, to estimate interaction energies of ligands with components of the PPARγ system and to substitute different ligands and examine receptor behaviors.

**Figure 6:**
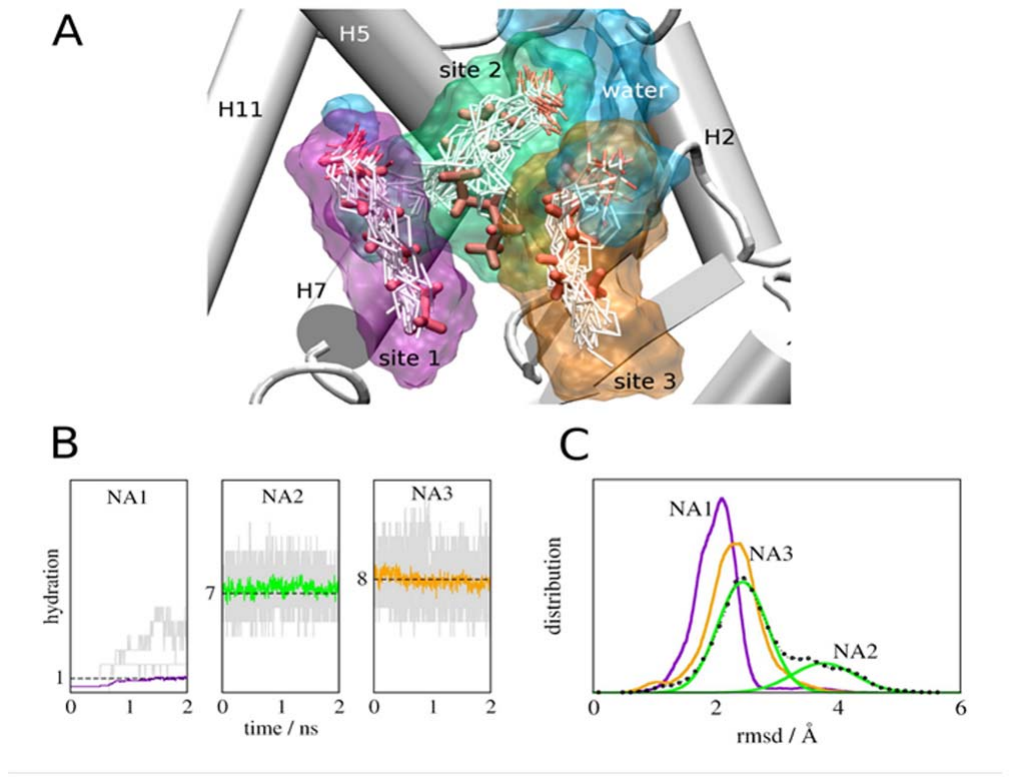
MD Simulations Predict Poor H12 packing in the presence of LA (C12). MD snapshot showing a hydrogen bond between Tyr473 and the ligand (cyan), which is persistent throughout the course of the simulations with NA (C9) and DA (C10), and an instantaneous conformation obtained from LA (C12) runs showing rupture of this bond and the concomitant displacement of H12 away from the body of the LBD (magenta).

We first performed MD simulations with the PPARγ+NA structure and modeled PPARγ structures in which DA (C10) or LA (C12) was substituted for NA in the tripartite binding mode to define relationships between MCFA chain length and PPARγ activity. We chose these MCFAs because, in our hands, DA exhibited high activity in transfections, whereas LA is weaker. Results suggest that NA (C9), DA (C10) and LA (C12) bind in the 3∶1 mode, but the former two MCFAs exhibit better fit in the PPARγ LBP than LA; LBP residues that comprise site I become more disordered in the presence of LA (Fig. S3). There is also a notable effect of MCFA chain length on H12 contacts (Figs. 6, S4); an important hydrogen bond contact between the MCFA polar carboxylate and the Tyr473 side chain is broken in LA simulations, but not DA simulations. This suggests that H12 is more stable in the presence of DA than NA. We propose this finding explains why LA exhibits reduced activity relative to DA and that this supports proposals that direct MCFA contacts with the inner surface of H12 are important in partial agonist activity [19].

### NA2 and NA3 Water Shells Play an Important Role in Binding

Since the β-sheet/H2-H3 region of the receptor appears preferentially stabilized in the presence of MCFAs, and NA2 and NA3 lie close to the inner surface of this region yet make few direct contacts with PPARγ protein (Fig. 1A), we analyzed interaction energetics of these MCFAs with LBP residues and ligand dynamics to understand how they may interact with the PPARγ LBD.

**Figure 7:**
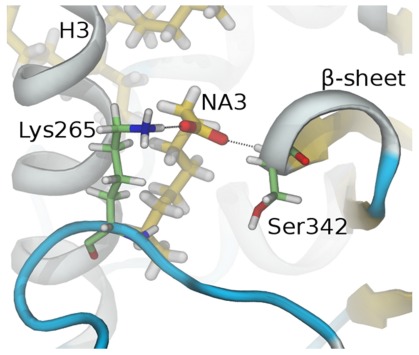
MD Simulations reveal a role for water in PPARγ-MCFA Interactions. (**A**) LBP showing superposed configurations of NA (white) observed in the simulations overlaid on native NA positions observed in the X-ray structure (red) and the corresponding average occupied volumes (color surfaces; NA1 is purple, NA2 is green and NA3 is orange. The average volume occupied by water molecules is shown in blue. Positions of NA carboxylate groups are marked with a C. (**B**) Waters surrounding NA at the three binding sites. The amount of water during independent simulations is shown in grey and average hydration numbers are shown in color; on average 1, 7, and 8 water molecules coordinate NA in sites I, II, and III respectively. (**C**) Distributions of RMSD of NA bound to PPARγ in the three binding sites computed from the simulations.

The simulations revealed unexpected aspects of MCFA binding. First, average binding energies of NA2 and NA3 with the PPARγ system are higher than NA1, despite fewer direct contacts of these ligands with protein, and this is related to hydration of the MCFA carboxylate group (Table 1, Fig. 7). Visualization of ligands reveals that NA1 (purple shell) interacts with small amounts of water (blue) throughout the simulation (Fig. 7A); on average less than one water lies near the NA1 carboxylate group (purple trace, Fig. 5B) although more waters (up to 5) can lie nearby at some instances (grey traces, Fig. 7B). By contrast, NA2 and NA3 carboxylates are continuously surrounded by large water pools (Fig. 7A) comprised of at least 7–8 waters (Fig. 7B, green and orange traces) with as many as 13–15 nearby in some frames (Fig. 7B). Second, NA2 and NA3 appear more flexible than NA1, judged by comparisons of initial NA position (Fig. 7A, red sticks) versus superposed conformations adopted in the simulation (white sticks) and differences in root mean squared displacements (RMSD) of ligand over the simulation (Fig. 7C and Fig. S4). In particular, the NA2 aliphatic chain (green; A2) fluctuates between two distinct average conformations; evidenced by the biphasic RMSD curve in Fig. 7C, and the NA3 carboxylate (C3) appears highly mobile (Fig. 7A). Inspection of the PPARγ+NA structure suggests that predicted differences in ligand mobilities are realistic; NA1 is well defined with low B-factors whereas NA2 and NA3 are poorly defined.

**Table 1:** Interaction energies of NAs with PPARγ system components.

	Interaction energies (kcal mol^−1^)		
MCFA	Complete system	PPARγ protein	Water
NA1	−140±14	−72±11	−67±18
NA2	−186±21	−22±12	−163±18
NA3	−188±24	−46±27	−141±23

**Figure 8:**
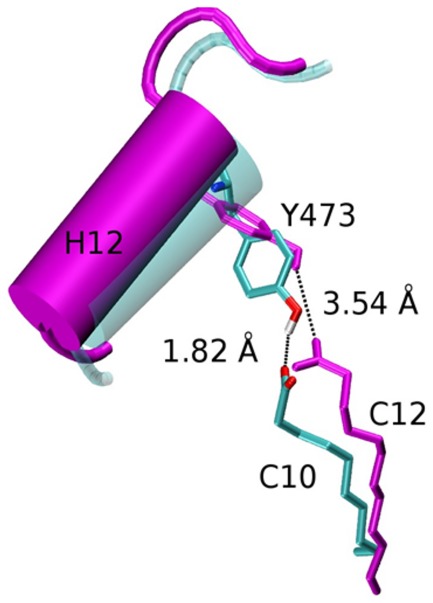
Predicted Interactions of NA3 and β-sheets. The figure shows a snapshot of the MD simulation, revealing contacts between NA3 and Ser342 that are not apparent in the crystal structure but are seen regularly throughout the course of the simulation.

Together, results suggest that pocket waters are important for MCFA binding; they bridge charged groups of the ligand to LBP polar residues (Discussion). Further, high NA2 and NA3 mobility means that both ligands can continuously form and break new contacts with LBP amino acids that are not always evident in the initial structure. Of interest (Fig. 8), the NA3 carboxylate engages in repeated contacts with Lys265 (H3) and Ser342 (β-sheets). It is interesting to suggest that these interactions could also help to stabilize the PPARγ β-sheets and H2-H3 region (Discussion).

## Discussion

In this study, we crystallized the PPARγ LBD without exogenously added ligand, but analysis of the resulting X-ray structure revealed MCFAs in the LBP, mostly NA (C9) with small amounts of OA (C8) as judged by mass spectroscopic analysis. The only plausible source of these MCFAs is the bacterial host used to express the protein. Thus, it is likely that our purification procedures did not strip MCFAs from the PPARγ preparations and the presence of these contaminating ligands increased PPAR stability and facilitated crystallization.

Our studies indicate that C8–C10 MCFAs are PPARγ partial agonists; this is in line with another study which shows that DA (C10) is a PPARγ modulator [19]. However, we also find that MCFAs exhibit differences in activity that are a function of both chain length and assay type and we propose that combined results of X-ray crystallography and MD simulations provide possible explanations for observed MCFA properties.

Our results suggest that chain length dependency of MCFA action relates to their ability to contact and stabilize the inner surface of H12 [19]. C8–C10 MCFAs are better PPARγ activators than C12–C14 MCFAs, see Fig. 3D and [22], with C10 displaying highest activity. Our MD simulations reveal more optimal contacts of C9–C10 MCFAs with Tyr473 on the inner surface of H12 than C12, which appears too long (Fig. 6 and S3). Also supporting the idea that MCFA contacts with H12 are important for activity is the organization in the crystallographic dimer; the PPARγ chain A (H12 active) contains three MCFAs in the LBP with one (NA1) in close juxtaposition to H12 whereas chain B (H12 inactive) contains two poorly defined MCFAs at the NA2 and NA3 positions which are not near the inner surface of H12. Our modeling also agrees with the proposal that LCFAs (C16 and upwards) are too large to bind the niche that is occupied by NA1 [19] and suggests that LCFAs will not be able to occupy the PPARγ LBP in a 3∶1 binding mode reported here (not shown) and must therefore bind the PPARγ LBP in a manner that differs from MCFAs.

In addition to a role for MCFA contacts with H12 in PPARγ activation, we noted surprising assay-specific differences in efficacy versus TZDs and we think that these features may be explained by the unique tripartite binding mode and differences in LBD surface stability versus TZDs. B-factor analysis reveals that MCFAs weakly stabilize H12 relative to TZDs (Fig. 5) and this correlates well with the observation that MCFAs are weak activators in the highly AF-2 dependent GAL-LBD assay (3–10% of TZD activity, Fig. 3). By contrast, MCFAs are stronger agonists in transfections with full length PPARγ; we do not completely understand this phenomenon but suggest that MCFAs affect PPARγ LBD activities that are important in the context of full length receptor. In this regard, MCFAs preferentially stabilize the loop between H11 and H12 and the β-sheet/H2-H3 region and the latter has been implicated in unexpected heterodimer contacts with the RXR DBD, revealed in the recent full length structure of a PPARγ/RXRα complex [25]. However, we recognize that other possible explanations for the relatively strong partial agonist activity of MCFAs at full length PPARγ; perhaps MCFAs alter cell behavior to enhance other aspects of PPARγ activity through secondary effects. Finally, MCFAs are effective inhibitors of cdk5 dependent phosphorylation at ser273 *in vitro* [10] and this correlates well with their ability to stabilize the β-sheet region, in common with other partial agonists [15] and selective PPAR modulators [10], [17].

Why do MCFAs preferentially stabilize the β-sheet/H2–H3 region relative to TZDs? At one level, the answer appears relatively simple; NA2 and NA3 occupy positions near the inner surface of this region whereas TZDs do not. We were puzzled by the fact that NA2 and NA3 do not appear to engage in large numbers of direct contacts with LBP residues in this region suggesting that these may be relatively weak interactions. However, our MD simulations indicate that NA2 and NA3 actually bind more tightly to the LBP than NA1 and that water molecules that bridge ligand carboxylate groups to polar LBP residues play an important role in binding affinity, similar to our proposed mechanism for TR subtype selective binding of the natural agonist Triac [26]. Strategies to enhance ligand-water contacts and ligand flexibility in this region of the PPARγ LBP could yield high affinity ligands that stabilize the β-sheet region.

Are MCFAs natural physiologically relevant PPARγ agonists? Studies of Malakapa et al. showed that diets containing decanoic acid or decanoic acid triglyceride improve insulin sensitivity in animal models [19]. It is also known that dietary MCFAs (usually as medium chain triglycerides) are abundant in certain foodstuffs, particularly milk, coconut and palm oil and dietary supplementation of these compounds improves aspects of metabolic syndrome and insulin resistance in humans [27]. Finally, our studies support those of previous papers which suggest that MCFAs are anti-adipogenic in cultured 3T3 cells (ref) and this property has also been observed *in vivo*. All of these findings are consistent with PPARγ partial agonism/antagonism and selective PPARγ modulation and, indeed, our results suggest that only MCFAs which bind PPARγ exhibit ant-adipogenic actions in 3T3 cells. While suggestive, much more work must be done to explore connections between physiologic actions and PPARγ binding. First, MCFAs used at high concentrations are likely to influence multiple metabolic pathways and regulatory events within the cell and it is difficult to parse actions that may be mediated through direct PPARγ binding from other effects on cell behavior; MCFAs may also reduce PPARγ protein and transcript levels by unknown indirect mechanisms [28]. Second, it is not clear whether MCFAs could reach sufficient concentrations to modulate PPARγ *in vivo*. Serum MCFA concentrations do reach the 100 µM-1 mM range [27], comparable to effective concentrations in transfections, and MCFAs are known to accumulate in adipocytes over time (OBESITY RESEARCH 2003); it will be important to explore connections between adipocyte FA content and PPARγ occupancy and binding.

Also of note is that the trimeric MCFA binding mode resembles aliphatic chain organization of triglycerides and phospholipids. Recent analysis of PPARα associated ligands in mouse liver indicates that the phospholipid 1-Palmitoyl-2-Oleoyl-sn-glycero-3-Phosphatidylcholine (16∶0/18∶1-GPC) is an endogenous PPARα activator [29]. Perhaps PPARγ may be able to accommodate phospholipids or triglycerides with MCFA moieties. More generally, our findings suggest that there may be natural ligands that behave as selective PPAR modulators with useful properties.

Finally, our results raise an obvious question; did PPARγ harbor bacterial ligands in previous “apo"- structures and could these have influenced PPARγ conformation? This was the case for PPARδ, where a reported apo-LBD structure was later shown to contain one long chain FA molecule in the LBP, predominantly cis-vaccenic acid (11, Z-octadecenoic acid), which stabilized PPARδ H12 in an active position [30], [31]. Additionally, bacterial phospholipids have been detected in LBPs of human liver receptor homolog 1 and steroidogenic factor 1 [32], [33] and long chain FAs co-purify with hepatocyte nuclear factor 4 [34]. For PPARγ, the LBD can be crystallized in true apo- form and we were mostly unable to find ligands in the LBP of previous apo-structures and have obtained our own structures of unliganded PPARγ LBDs and cannot detect MCFAs or other ligands in LBPs. We did find one possible instance of an FA-like electron density that resembles a long chain polyunsaturated FA in the original apo-PPARγ structure [18] (Fig. S5). Given that bacterial ligands have now co-crystallized with multiple NRs, it will be very important to consider the possible presence of bacterial ligands in “apo"-PPARγ and-NR structures and the potential impact of such ligands on LBD conformation.

## Materials and Methods

### PPARγ-LBD Expression and Purification

PPARγ (204–477) was expressed as an N-terminal His-tagged protein using pET28a vector (Novagen), as previously described [35]. Freshly transformed *E. coli* BL21 (DE3), obtained from Novagen, USA, were grown in LB media at 20°C to an OD_600_ = 1.2. The culture was induced (1 mM IPTG) and grown at 20°C for 5 hr. Cells were resuspended in a 25 ml/L culture of buffer A (5 mM imidazole, 25 mM Tris, 100 mM NaCl, 1 mM TCEP, pH8.0) and disrupted by lysozyme treatment followed by sonication and the soluble fraction isolated by centrifugation (35,000×g, 45 min). Supernatant was loaded onto Co^2+^-charged resin TALON (BD Biosciences), washed with 20 column volumes buffer A and eluted with buffer B (500 mM imidazole, 25 mM Tris, 100 mM NaCl, 1 mM TCEP, pH8.0). The fraction with protein was dialyzed over buffer C (25 mM Tris, 50 mM NaCl, 1 mM TCEP, pH8.0) to remove imidazole, and protein cleaved with thrombin (Sigma-Aldrich) (10 U/mg) at room temperature for 12 h. PPARγ-LBD was quantified using the Bradford protein assay (Pierce) and Coomassie Blue staining.

### Crystallization and Structure Determination

PPARγ crystals grew in hanging drop crystallization trials. 2 ml of well solution containing 0.1 M Tris-HCl, pH 7.5+0.9 M sodium citrate were equilibrated vs. 2 µl concentrated protein solution. Crystals were obtained after 3–5 days at 18°C. Prior to data collection, a single crystal was immersed in cryoprotectant containing 20% glycerol and flash frozen in a nitrogen stream at −100°C. X-ray diffraction data were collected at the protein crystallography W01B-MX2 beamline of the Brazilian Synchrotron Light Laboratory (LNLS), Campinas, Brazil [36]. Observed reflections were integrated, merged, and scaled with DENZO and SCALEPACK in HKL2000 [37]. The structure was solved by molecular replacement using PHASER [38] and a previously published PPARγ LBD structure (PDB code: 1ZEO [39]) as the search model. PHENIX [40] was used for structural refinement with several cycles of model rebuilding in COOT [41]. The coordinates and structure factors of PPARγ-NA and PPARγ-Rosiglitazone have been deposited in the Protein Data Bank with the PDB ID codes 4EM9 and 4EMA, respectively.

### Cell Culture and Transfection

Transfections (HeLa or HepG2 cells, obtained from American Type Culture Collection, Manassas, VA; 5XGAL4 RE or DR1 luciferase reporter) used +/− GAL-PPAR LBD or full length PPARγ expression vector. Luciferase assays were performed by standard methods, standard errors were derived from quadruplet points and experiments repeated >3 times. PPARγ mutants were created using the Stratagene kit and verified by sequence analysis. For NIH3t3 differentiation assays, cells were cultured in standard FBS supplemented with Rosi or MCFA [23].

### 3T3-L1 Differentiation Assay and Oil Red O staining

Murine 3T3-L1 cells were maintained in Preadipocytes medium (Zen-Bio). Cells were induced to differentiate two days post confluent using DMEM/Ham's F-12 medium supplemented with Insulin, Dexamethasone and Isobutylmethylxanthine in the absence or presence of 100 nM Rosiglitasone, 1 mM HA C6, or 1 mM DA C10 as indicated in Figure legend. Cells were then fed with Zen Bio's AM-1-L1 media. On day 7 cells were fixed, stained with Red Oil O and phase contrast images were taken using an Olympus Ix81 microscope (10× magnification).

### Molecular dynamics

MD simulations used the PPARγ chain A X-ray structural model. The missing loop (262–273) was modeled from residues 257–277 of a previous structure (PPARγ 1PRG model [18]), which fit well into the structure after alignment with LovoAlign [42]. A solvation shell of at least 15 Å was created using VMD [43] and Sodium and Chloride ions added in a concentration close to 0.15 mol L^−1^ to render the system electrically neutral. The final system contained 53,530 atoms. Simulations were performed with NAMD [44] using periodic boundary conditions and CHARMM parameters [41] for protein and NA (C9) and TIP3P [42] parameters for water. Auxiliary simulations were also performed for DA and LA and initial structures were modeled from the NA-PPARγ crystal structure by adding missing atoms to C9.

A 12 Å cutoff radius was used for van der Waals interactions, whereas the electrostatic forces were handled by Particle Mesh Ewald sums [43]. Temperature was set to 300 K and pressure to 1 atm in all simulations. A 2 fs time-step was used integrate the equations of motions using the Verlet algorithm. 12 independent sets of equilibration and production simulations were performed. The protocol for each equilibration/simulation was: (1) Energy minimization using 500 steps of conjugate gradients (CG), keeping all atoms fixed, except modeled loop. (2) 2000 CG steps keeping only protein atoms fixed except modeled loop. (3) With same atoms fixed, 200 ps MD in the NPT ensemble, using temperature scaling at every 1 ps and a Langevin piston to control pressure with a period of 0.2 ps and damping time of 0.1 ps. (4) 500 CG steps followed by 150 ps MD with the same protocol, removing restraints on all but fixed Cα atoms. (5) 200 ps MD with the same protocol, without restraint. (6) Production runs started from the last frame of this equilibration simulation and were 2 ns long. The same protocol was used for production runs, except that temperature was controlled via a Langevin bath with a damping coefficient of 1 ps^−1^.

## Supporting Information

Figure S1 **Structures of PPARγ A and B chains.** The left (copper) shows the organization of the PPARγ dimer B-chain, with positions of MCFAs marked in purple. The right figure shows overlays of PPARγ A (green) and B (copper) chains; note the different position of C-terminal H12.
(TIF)

Figure S2 **Mass spectroscopic analysis of MCFA interactions with PPARγ LBD.** We performed MS analysis of purified PPARγ preparation used for crystallization. MS spectra of the derivatized MCFAs OA (top) and NA (bottom) analyzed by GC/MS are shown. Analysis of extracts and FA Methyl Ester standards (FAMEs; C8:0-C12:0; C13:0-C17, Sigma Chem. Co, and C18:0-C20:5 RESTEK; Bellefonte, PA, USA) were performed on a GC-MS system Shimadzu, mod. QP5000, fitted with an FID and a split/splitless injector. Separations were performed on a RESTEK Rtx-wax capillary column [15 m, 0.25 mm i.d., 0.25 mm film thickness] (Bellefonte, PA, USA) connected to the MS ion source and helium was used as the carrier gas (1.5 ml/min). Oven temperature was maintained at 80°C for 3 min, then increased at 3°C/min to 250°C and stabilized until all components eluted. The ion source (Electron Impact – EI) was kept at 200°C and the transfer line at 310°C. EI spectral (70 eV) analyses were acquired with a mass selective detector (MSD). Data acquisitions were performed using Class-VP 4.3 software (Shimadzu, Japan). Standards were analyzed by injecting 0.4 ml of a solution of FAMEs (1∶10 v/v in hexane) with a split ratio 1∶50, while esterified extracts were analyzed by injecting 2 µL (3.2 mg of lipid material). FAs were identified by comparison between their retention times with FAME standards during GC analysis and matching mass spectra for samples and standards. A compound was identified if its retention time and EI mass spectrum were identical with reference compound. FAMEs of the web FAs were obtained by transesterification with a solution of H_2_SO_4_ 10% in methanol, at 120°C during 90 min.
(TIF)

Figure S3 Effects of different MCFAs on the PPAR LBP. RMSD distribution of the BP residues comprising sites I, II, and III, relative to the C9-PPARγ holo crystal structure reported here, from simulations with C9, C10, and C12. The distributions are unimodal for C10-bound LBD (red), suggesting a snuggled fit of this ligand in the BP. The simulations also suggest that the BP presents largest conformational variations in the presence of C12 (cyan). This is particularly noticeable for residues comprising binding site I near H12.
(TIF)

Figure S4 RMSD distribution for the Tyr473 residue in H12 computed for C9, C10, and C12 MCFAs bound to PPARγ. Y473 is least mobile in the presence of C10 and most mobile with C12, where it exhibits a biphasic distribution consistent with two average positions.
(TIF)

Figure S5 **Possible occupancy of LBP in a previous Apo-PPARγ Structure.** **A**) The figure shows electron density calculated from structural factors deposited for PDB structure 1PRG (left). **B**) Superposition with crystal structure of PPAR bound to 5,8,11,14,17-eicosapentaenoic acid (EPA, PDB 3GWX) shows a significant degree of correlation between the experimental electron density and the bound fatty acid (right).
(TIF)

Table S1 **Data collection and refinement statistics.**
(DOCX)
