# Supplementary figures and images for: Medium Chain Fatty Acids Are Selective Peroxisome Proliferator Activated Receptor (PPAR) γ Activators and Pan-PPAR Partial Agonists
Source: PLoS One. 2012 May 23;7(5):e36297. doi: 10.1371/journal.pone.0036297 (PMC3359336; doi:10.1371/journal.pone.0036297)

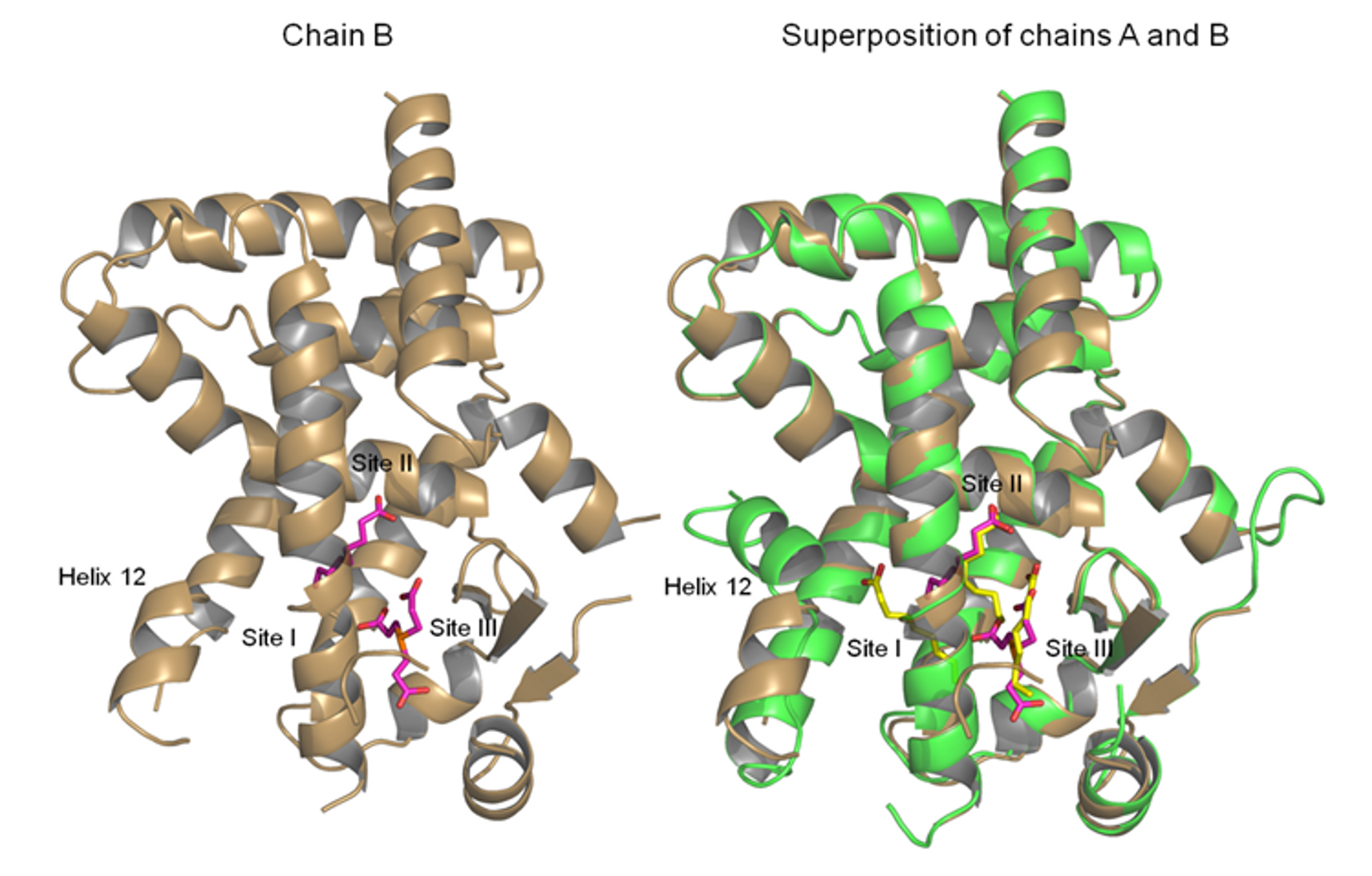

Supplement: Figure S1 — Structures of PPARγ A and B chains. The left (copper) shows the organization of the PPARγ dimer B-chain, with positions of MCFAs marked in purple. The right figure shows overlays of PPARγ A (green) and B (copper) chains; note the different position of C-terminal H12. (TIF) [file pone.0036297.s001.tif]

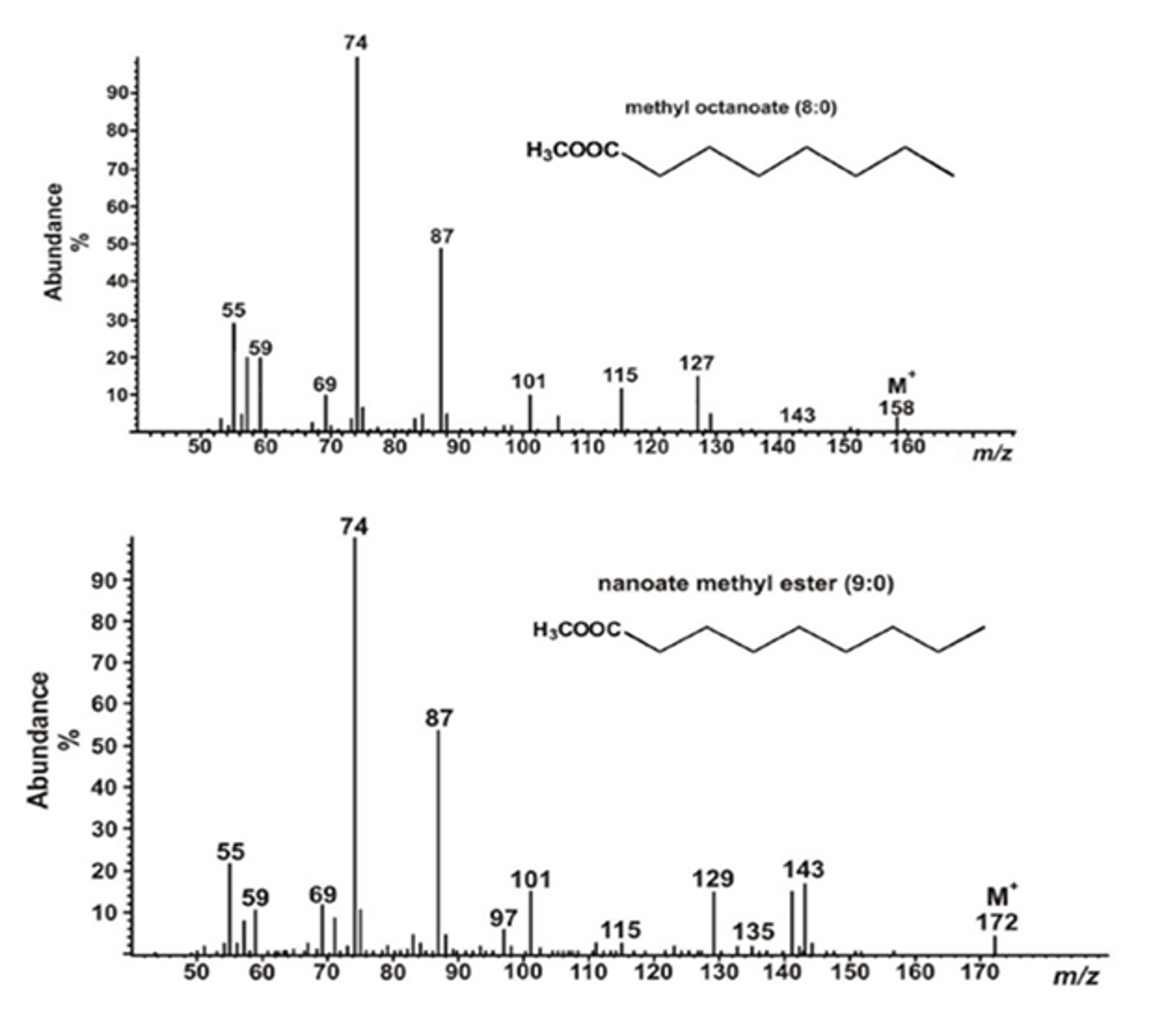

Supplement: Figure S2 — Mass spectroscopic analysis of MCFA interactions with PPARγ LBD. We performed MS analysis of purified PPARγ preparation used for crystallization. MS spectra of the derivatized MCFAs OA (top) and NA (bottom) analyzed by GC/MS are shown. Analysis of extracts and FA Methyl Ester standards (FAMEs; C8:0-C12:0; C13:0-C17, Sigma Chem. Co, and C18:0-C20:5 RESTEK; Bellefonte, PA, USA) were performed on a GC-MS system Shimadzu, mod. QP5000, fitted with an FID and a split/splitless injector. Separations were performed on a RESTEK Rtx-wax capillary column [15 m, 0.25 mm i.d., 0.25 mm film thickness] (Bellefonte, PA, USA) connected to the MS ion source and helium was used as the carrier gas (1.5 ml/min). Oven temperature was maintained at 80°C for 3 min, then increased at 3°C/min to 250°C and stabilized until all components eluted. The ion source (Electron Impact – EI) was kept at 200°C and the transfer line at 310°C. EI spectral (70 eV) analyses were acquired with a mass selective detector (MSD). Data acquisitions were performed using Class-VP 4.3 software (Shimadzu, Japan). Standards were analyzed by injecting 0.4 ml of a solution of FAMEs (1∶10 v/v in hexane) with a split ratio 1∶50, while esterified extracts were analyzed by injecting 2 µL (3.2 mg of lipid material). FAs were identified by comparison between their retention times with FAME standards during GC analysis and matching mass spectra for samples and standards. A compound was identified if its retention time and EI mass spectrum were identical with reference compound. FAMEs of the web FAs were obtained by transesterification with a solution of H2SO4 10% in methanol, at 120°C during 90 min. (TIF) [file pone.0036297.s002.tif]

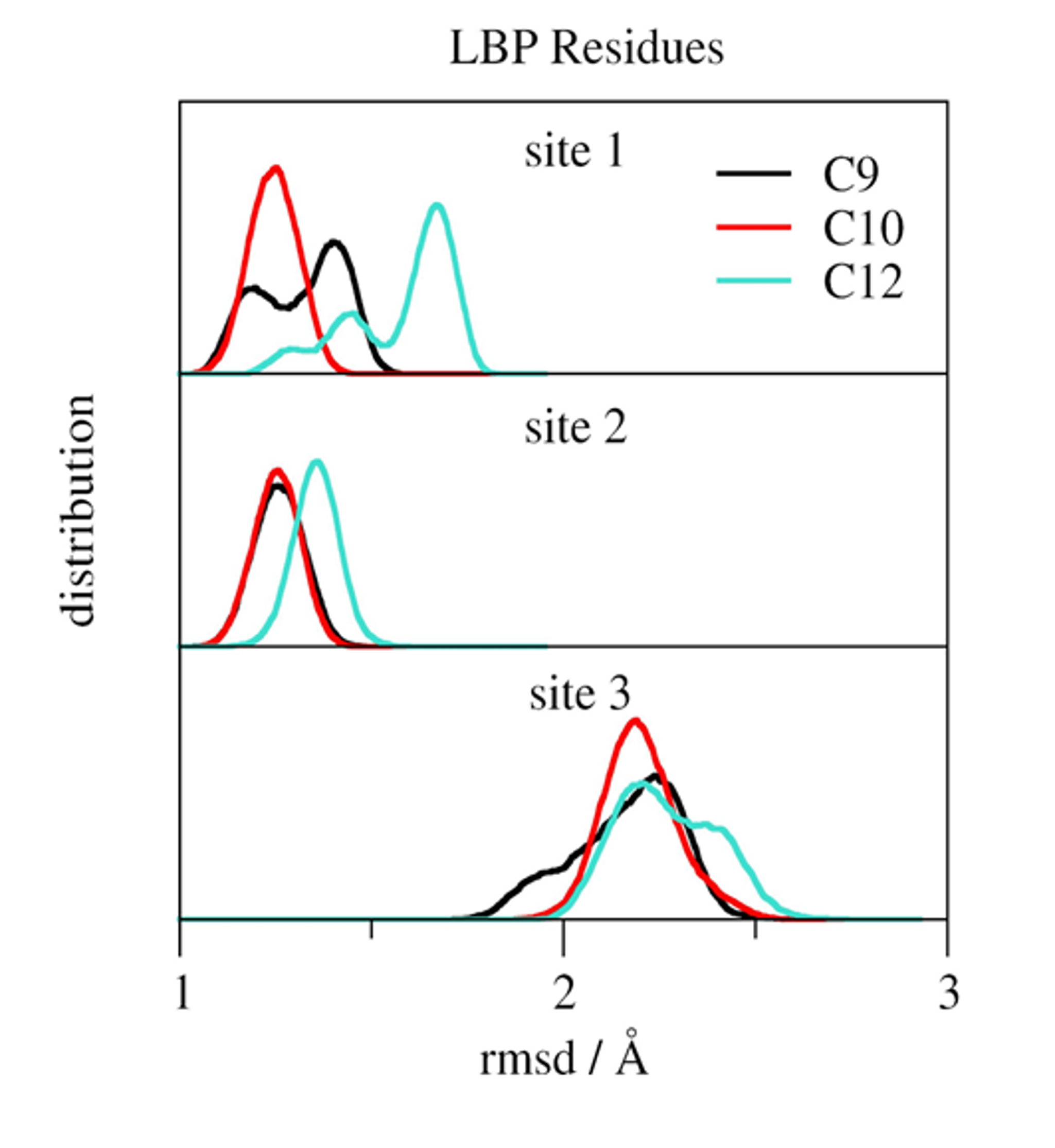

Supplement: Figure S3 — Effects of different MCFAs on the PPAR LBP. RMSD distribution of the BP residues comprising sites I, II, and III, relative to the C9-PPARγ holo crystal structure reported here, from simulations with C9, C10, and C12. The distributions are unimodal for C10-bound LBD (red), suggesting a snuggled fit of this ligand in the BP. The simulations also suggest that the BP presents largest conformational variations in the presence of C12 (cyan). This is particularly noticeable for residues comprising binding site I near H12. (TIF) [file pone.0036297.s003.tif]

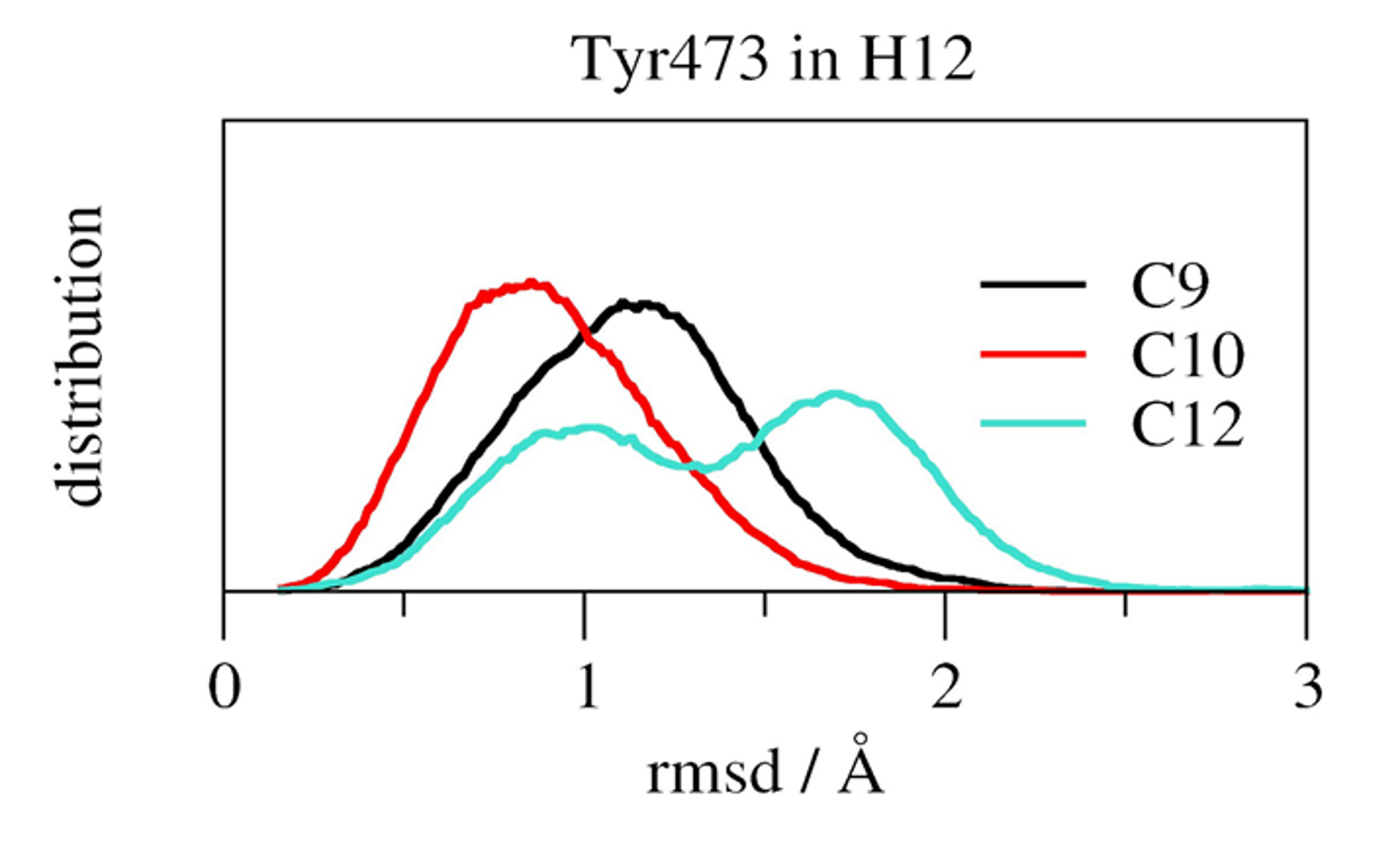

Supplement: Figure S4 — RMSD distribution for the Tyr473 residue in H12 computed for C9, C10, and C12 MCFAs bound to PPARγ. Y473 is least mobile in the presence of C10 and most mobile with C12, where it exhibits a biphasic distribution consistent with two average positions. (TIF) [file pone.0036297.s004.tif]

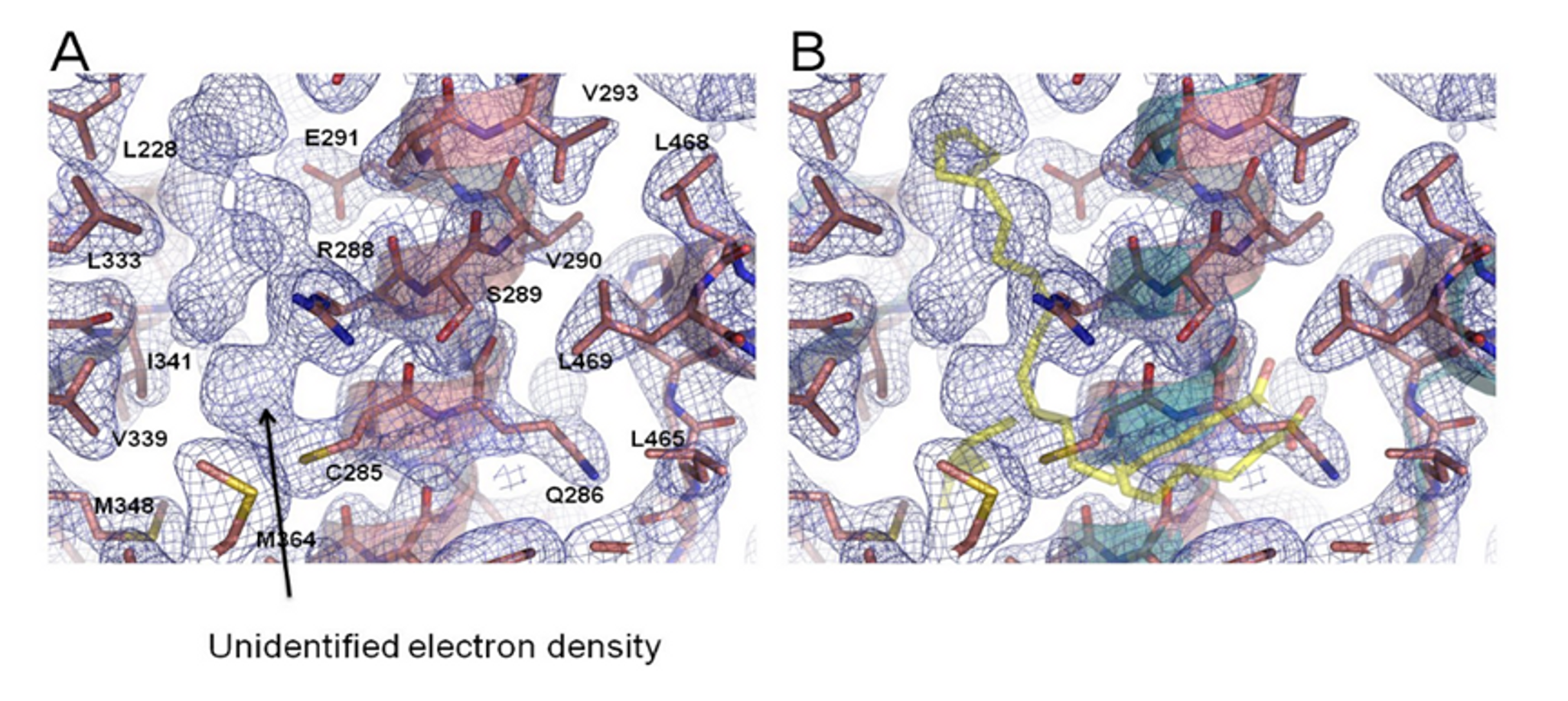

Supplement: Figure S5 — Possible occupancy of LBP in a previous Apo-PPARγ Structure. A) The figure shows electron density calculated from structural factors deposited for PDB structure 1PRG (left). B) Superposition with crystal structure of PPAR bound to 5,8,11,14,17-eicosapentaenoic acid (EPA, PDB 3GWX) shows a significant degree of correlation between the experimental electron density and the bound fatty acid (right). (TIF) [file pone.0036297.s005.tif]
