# Supplementary material for: Medium Chain Fatty Acids Are Selective Peroxisome Proliferator Activated Receptor (PPAR) γ Activators and Pan-PPAR Partial Agonists
Source: PLoS One. 2012 May 23;7(5):e36297. doi: 10.1371/journal.pone.0036297 (PMC3359336; doi:10.1371/journal.pone.0036297)
Supplement: Table S1 — Data collection and refinement statistics. (DOCX) [file pone.0036297.s006.docx]

**Supplementary** **Table 1**

| Data collection and refinement statistics | | | | |
| --- | --- | --- | --- | --- |
| **Diffraction data** | | | | |
| **Cristal** | | ***PPARγ-NA*** | ***PPARγ-Rosiglitazone*** | |
| **Source** | | Syncrotron | Syncrotron | |
| **Wavelength (Å)** | | 1.46 | 1.46 | |
| **Space group** | | C2 | C2 | |
| **Lattice parameters (Å)** | | a = 92.73, b = 62.25,  c = 118.64,  β = 101.94 | a = 90.51, b = 61.95,  c = 117.60,  β = 100.70 | |
| **Resolution (Å)** | | 50-2.09 (2.17-2.09) | 28.89-2.54 (2.66-2.54) | |
| **Completeness (%)** | | 94.0 (59.9) | 98.23 (91.7) | |
| **I / σ(I)** | | 24.86 (2.23) | 12.66 (2.42) | |
| **Redundancy** | | 5.6 (3.3) | 5.5 (4.3) | |
| **R-merge linear/square** | | 0.05/0.05 (0.35/0.22) | 0.06/0.06 (0.57/0.47) | |
| **Refinement Statistics** | | | |  |
| **Resolution (Å)** | | 2.10 | 2.54 | |
| **No. reflections** | | 36859 | 20835 | |
| **Rwork (%)** | | 20.49 | 17.98 | |
| **Rfree (%)** | | 23.51 | 22.47 | |
| **No. Atoms** | | 4382 | 4102 | |
|  | ***Protein*** | 3993 | 3998 | |
|  | ***Heterogen*** | 78 | 25 | |
|  | ***Solvent*** | 311 | 79 | |
| **B-factor** |  | 55.34 | 60.34 | |
|  | ***Protein*** | 54.83 | 60.36 | |
|  | ***Heterogen*** | 71.74 | 68.05 | |
|  | ***Solvent*** | 61.92 | 59.12 | |
| **Rmsd bond (Å)** | | 0.005 | 0.011 | |
| **Rmsd angles (º)** | | 0.76 | 1.583 | |
| Values in parentheses are for highest-resolution shell | | | | |
